# Clathrin mediates both internalization and vesicular release of triggered T cell receptor at the immunological synapse

**DOI:** 10.1073/pnas.2211368120

**Published:** 2023-02-02

**Authors:** Audun Kvalvaag, Salvatore Valvo, Pablo F Céspedes, David G Saliba, Elke Kurz, Kseniya Korobchevskaya, Michael L Dustin

**Affiliations:** ^a^Nuffield Department of Orthopaedics, Rheumatology and Musculoskeletal Sciences, Kennedy Institute of Rheumatology, University of Oxford, Oxford OX3 7FY, UK; ^b^Department of Molecular Cell Biology, Institute for Cancer Research, Oslo University Hospital, Montebello, Oslo 0379, Norway; ^c^Department of Applied Biomedical Science, Faculty of Health Science, University of Malta, Msida MSD 2080, Malta

**Keywords:** clathrin, synapse, receptors, endocytosis, ectocytosis

## Abstract

Ligation of T cell receptor (TCR) to peptide–MHC (pMHC) complexes initiates signaling leading to T cell activation and TCR ubiquitination. Ubiquitinated TCR is then either internalized by the T cell or released toward the antigen-presenting cell (APC) in extracellular vesicles. How these distinct fates are orchestrated is unknown. Here, we show that clathrin is first recruited to TCR microclusters by HRS and STAM2 to initiate release of TCR in extracellular vesicles through clathrin- and ESCRT-mediated ectocytosis directly from the plasma membrane. Subsequently, EPN1 recruits clathrin to remaining TCR microclusters to enable trans-endocytosis of pMHC–TCR conjugates from the APC. With these results, we demonstrate how clathrin governs bidirectional membrane exchange at the immunological synapse through two topologically opposite processes coordinated by the sequential recruitment of ecto- and endocytic adaptors. This provides a scaffold for direct two-way communication between T cells and APCs.

## Significance

Clathrin is well known for its role in clathrin-mediated endocytosis, one of the most extensively studied cell biological processes. Here, we describe a separate role for clathrin in regulating release of T cell receptor (TCR) loaded vesicles directly from the plasma membrane, and we show that clathrin in fact regulates both TCR loaded vesicle release and pMHC-conjugated TCR internalization. This choice of which direction the TCR moves is coordinated by the temporally dynamic recruitment of clathrin adaptor proteins. Initially, clathrin is recruited to TCR microclusters by the ESCRT-0 components HRS and STAM2 to induce release of TCR in extracellular vesicles. Subsequently, the endosomal clathrin adaptor EPN1 takes over and initiates clathrin-mediated trans-endocytosis of TCR and associated pMHC.

The fundamental molecular interactions responsible for regulating the adaptive immune response occur within a nanoscale gap between T cells and antigen-presenting cells (APCs) termed the immunological synapse (IS). IS formation is induced upon T cell receptor (TCR) interactions with agonist peptide-Major Histocompatibility Complex (pMHC) on the surface of APCs (1, 2). This process can be recapitulated by antigen presentation on supported lipid bilayers (SLBs), a minimal system composed of a mobile lipid phase configured to present relevant ligands at physiological densities (3, 4). Such IS formation on SLBs allows for microscopic analysis of the receptor–ligand interactions and membrane trafficking events underlying the initiation and effector functions of the adaptive immune system.

During activation, the components of the IS rearrange in the opposing lipid membranes to form a characteristic bull’s-eye pattern consisting of three primary domains (5, 6). The bull’s-eye itself is termed the central supramolecular activation cluster (cSMAC) and is dominated by TCR and its ligands in a synaptic cleft. This area is surrounded by an adhesive ring defined by Lymphocyte Function-associated Antigen-1 (LFA-1) on the T cell side associated with intercellular adhesion molecule-1 (ICAM-1) on the SLB termed the peripheral supramolecular activation cluster (pSMAC). The outer edge of the contact is defined by an F-actin–rich sensory compartment termed the distal supramolecular activation cluster (dSMAC). TCR engagement is initiated in microclusters that arise from filopodia in the dSMAC and retain protrusive activity even as they traffic through the pSMAC toward the cSMAC (7–11). This is accompanied by CD3 tyrosine phosphorylation, recruitment of Zeta-associated protein of 70 kDa (ZAP-70), and phosphorylation of Linker of Activated T cells (LAT) (12) and recruitment of SH2-domain-containing leukocyte protein of 76 kDa (SLP-76) (13). These then form condensates organized by LAT, traversing concentric actin networks en route to the cSMAC (14).

Formation of the cSMAC by helper T cells has been shown to require recognition of ubiquitinated TCR by Tumor susceptibility gene 101 (TSG101), a component of the endosomal sorting complex required for transport (ESCRT) (15). Then, following TSG101-dependent TCR sorting, the ESCRT-associated ATPase VPS4 mediates scission of TCR loaded extracellular vesicles termed synaptic ectosomes, which bud directly from the plasma membrane into the synaptic cleft (16). This is mechanistically similar to formation of the intraluminal vesicles (ILVs) of multivesicular endosomes and budding of HIV virions from the plasma membrane (17, 18). Prior to action of TSG101, the ESCRT component Hepatocyte growth factor-regulated tyrosine kinase substrate (HRS) recognizes ubiquitinated cargo and recruits clathrin, which assembles into flat lattices to mediate recruitment of subsequent ESCRT machinery (19, 20). Clathrin- and HRS-positive vesicles have also been shown to polarize toward the IS during T cell activation, and they have been implicated in recruiting F-actin there (21). However, it is not known whether clathrin is involved in the formation of synaptic ectosomes.

Clathrin is primarily known for its role in endocytosis, where it is recruited to the plasma membrane by adaptor proteins such as Epsin-1 (EPN1) and Adaptor Protein complex-2 (AP-2) (22, 23). Together, these proteins facilitate deformation and invagination of the membrane which is ultimately pinched off as a clathrin-coated endocytic vesicle (24) by the large GTPase dynamin (25–27). Previous reports have shown that this mechanism is engaged to internalize TCR during constitutive TCR recycling (28, 29) and to internalize nonactivated bystander TCR during T cell activation (30). It has also been shown that TCR triggering leads to phosphorylation of clathrin heavy chain (CHC), (31) and TCR has been observed in clathrin-coated pits following antibody activation (32). However, conflicting evidence has indicated that clathrin is not involved in internalization of triggered TCR in the Jurkat cell line (30, 33, 34). Hence, the role of clathrin in endocytosis of pMHC–TCR conjugates is yet to be established.

Synaptic ectosomes are important for delivery of T cell help through CD40 ligand and additional signals (35). TCR endocytosis is required for postendocytic signaling and regulation of pMHC availability (36, 37). Therefore, it is critical to understand the balance between ectocytosis and endocytosis of the TCR. Here, we show that clathrin is pivotal during T cell activation through its essential role in two sequential processes. First, clathrin is essential for ESCRT-mediated release of TCR loaded synaptic ectosomes at the cSMAC. As the IS matures, there is a temporal switch from the clathrin-associated ESCRT components HRS and STAM2 to the endocytic clathrin adaptor EPN1. Remaining antigen-ligated TCRs are then internalized by clathrin-mediated trans-endocytosis.

## Results

### Clathrin Is Recruited to TCR Microclusters at the IS.

**Fig. 1.**
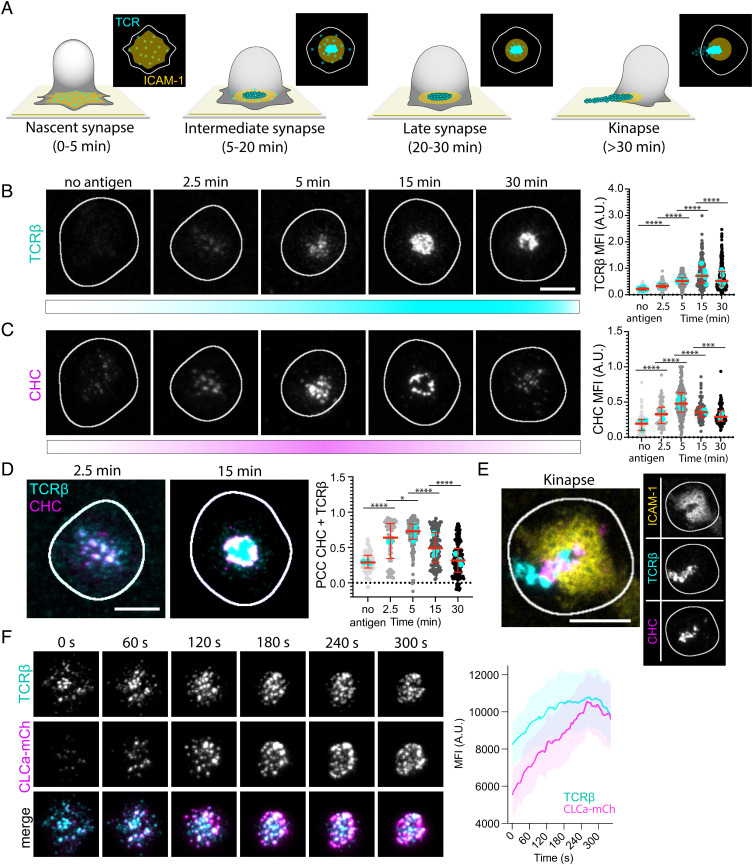
Clathrin is recruited to the IS. (*A*) Schematic of the different maturation stages of the IS formed between a T cell and an SLB. ICAM-1 (yellow) forms the adhesion ring, and TCRβ microclusters (cyan) reach the center of the contact area where some are released as synaptic ectosomes that are left behind as symmetry breaking allows the T cell to relocate. (*B* and *C*) Representative TIRF micrographs of AND mCD4 T cells incubated on SLBs either with ICAM-1-AF405 (200/µm^2^) alone for 5 min or with ICAM-1-AF405 + I-E^k^-MCC (20/µm^2^) for 2.5, 5, 15, and 30 min and labeled with anti-mouse TCRβ and anti-CHC. N_cells_ ≥ 62 per time point. (Scale bar, 5 µm.) The *Right* panels are quantifications of the MFI of TCRβ and CHC across the synaptic interface. Lines are median value ± IQR, and cyan dots are average values from individual experiments. (*D*) Representative TIRF micrographs emphasizing the colocalization between CHC (magenta) and TCRβ (cyan) at 2.5 min and 15 min. The *Right* panel is quantification of the PCC between CHC and TCRβ across the synaptic interface from the micrographs in *B*–*D*. (*E*) Representative TIRF micrograph of a kinapse formed by an AND T cell incubated for 30 min on an SLB with ICAM-1-AF405 (yellow) + IE^k^-MCC as before. Note how CHC (magenta) is overlapping with TCRβ (cyan) in the region where the pSMAC is broken. (*F*) Representative time frames from a movie of a live mCD4 AND T cell expressing CLCa-mCherry (magenta) while forming an IS on an SLB with ICAM-1-AF405 (200/µm^2^) and I-E^k^-MCC (50/µm^2^). The TCR is labeled with anti-TCRβ (cyan). The *Right* panel is mean temporal fluorescence intensity traces ± SEM of TCR microclusters with overlapping CLCa-mCherry fluorescence. N_cells_ = 5.

The IS undergoes several stages of maturation during T cell activation on SLBs, as illustrated in Fig. 1*A* [*Insets* depict TCR microcluster formation and movement at the SLB as seen by an inverted microscope (16)]. The process is initiated during presynaptic interactions of TCR at the tips of microvilli with anti-CD3 or pMHC on the SLB leading to rapid activation of LFA-1–ICAM-1–mediated adhesion and immediately followed by spreading of the T cell with the formation of LFA-1 and TCR microclusters (0 to 5 min) (4, 8, 38–41). LFA-1 and TCR then move centripetally in the nascent IS and accumulate in the pSMAC and cSMAC, respectively, of the maturing IS (5 to 20 min). After about 20 to 30 min, signaling is sustained by a minimal number of TCR microclusters and formation of a polarized motile profile referred to as a kinapse is often observed (>30 min) (42, 43).

We first asked whether clathrin is recruited to TCR microclusters at any stage during IS formation. Monoclonal T cells from AND TCR transgenic mice specific for I-E^k^ with a moth cytochrome C peptide 88-103 (I-E^k^-MCC) were incubated on SLB with ICAM-1-AF405 (200 µm^−2^) alone for 5 min or with ICAM-1 plus I-E^k^-MCC (20 µm^−2^) for 2.5, 5, 15, and 30 min. We then fixed the T cells and immunolabeled the TCR with the anti-mouse TCRβ H57 Fab tagged with Alexa Flour 488 (TCRβ) and clathrin with an anti-CHC antibody. By imaging the cells by total internal reflection fluorescence (TIRF) microscopy, we observed formation of TCR microclusters after 2.5 min in the presence of antigen and an increase in TCRβ fluorescence intensity compared to no antigen (Fig. 1*B*) [All raw image data are available at OSF.IO/SXEQR]. The TCRβ intensity peaked after 15 min before decreasing by about 50% after 30 min. Intriguingly, the increase in TCRβ intensity after 2.5 min was accompanied by a twofold increase in CHC fluorescence intensity (Fig. 1*C*). CHC and TCR were extensively colocalized with Pearson correlation coefficients (PCCs) of 0.64 at 2.5 min and 0.73 at 5 min [the PCC ranges from 1 (perfect correlation) to −1 (anticorrelation)]. The CHC intensity peaked after 5 min and declined as the IS matured and TCR accumulated in the cSMAC. After 15 min, clathrin appeared to be confined to the cSMAC periphery, and at 30 min, clathrin was no longer strongly associated with TCR in the cSMAC (Fig. 1*D*). A fraction of the cells had already formed kinapses at this time point. These are defined by a broken pSMAC and a trail of released TCR loaded vesicles being left behind as the cell starts to migrate. Surprisingly, the break in the pSMAC was crowded by clathrin (Fig. 1*E*). This appeared to overlap with TCR still associated with the plasma membrane which might indicate that clathrin is mediating T cell disengagement from its substrate by either internalizing or releasing pMHC-conjugated TCR.

Live imaging of T cells with mCherry-tagged clathrin light chain A (CLCa-mCherry) (44) revealed that recruitment of TCR and associated CLCa increased at the IS as microclusters moved centripetally (Fig. 1*F* and Movie S1). Also, by increasing the I-E^k^-MCC density on the SLB, we observed that the CHC intensity increased accordingly (*SI Appendix*, Fig. S1*A*). We next applied TIRF combined with structured illumination microscopy (TIRF–SIM) with up to 90 nm lateral and 100 nm axial resolution to investigate temporal clathrin recruitment to TCR microclusters in human CD4 (hCD4) T cells on SLBs with ICAM-1 and an agonistic anti-CD3ε Fab UCHT1 (*SI Appendix*, Fig. S1*B*). We then observed CHC recruitment to TCR microclusters as these coalesced into large membrane domains within 5 min of activation. After 15 min, the TCR- and clathrin-enriched membrane domains appeared to split into smaller domains which after 30 min appeared as punctate TCR structures likely representing extracellular vesicles, with CHC only colocalizing with TCR at the cSMAC periphery.

We conclude that clathrin is recruited to TCR microclusters as they move to the center of the nascent IS but that it is largely excluded from the released vesicles at the center of the mature cSMAC.

### Clathrin Adaptors Recruited to the IS Undergo a Temporal Shift from the ESCRT-0 Components HRS and STAM2 to the Endocytic Adaptor EPN1.

Previous reports have shown that the ESCRT-I component TSG101 is required for cSMAC formation, whereas the ESCRT-associated ATPase VPS4 is required for scission of the bud neck of TCR loaded nascent synaptic ectosomes (15, 16). This process is analogous to ESCRT-mediated formation of ILVs in which TSG101 is recruited to flat clathrin lattices on the limiting endosomal membrane by HRS and STAM2 (20). We therefore asked whether a similar mechanism was taking place at the IS.

**Fig. 2.**
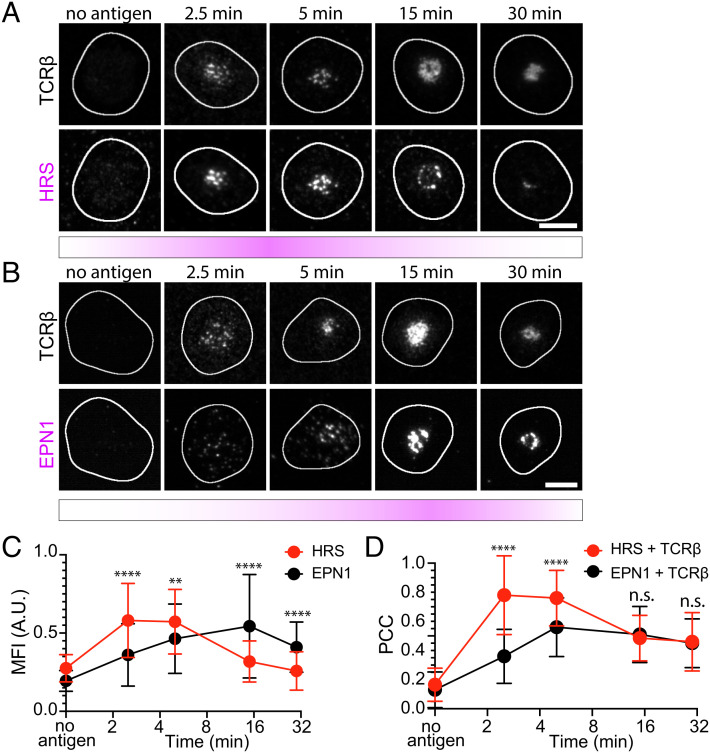
HRS and EPN1 are recruited to the IS at different stages of synapse maturation. (*A*) Representative TIRF micrographs of AND mCD4 T cells incubated on SLBs either with ICAM-1-AF405 (200/µm^2^) alone for 5 min or with ICAM-1-AF405 + I-E^k^-MCC (20/µm^2^) for 2.5, 5, 15, and 30 min and labeled with anti-mouse TCRβ and anti-HRS. N_cells_ ≥ 65 per time point. (Scale bar, 5 µm.) (*B*) Representative TIRF micrographs of AND mCD4 T cells incubated on SLBs either with ICAM-1-AF405 (200/µm^2^) alone for 5 min or with ICAM-1-AF405 + I-E^k^-MCC (20/µm^2^) for 2.5, 5, 15, and 30 min and labeled with anti-mouse TCRβ and anti-EPN1. N_cells_ ≥ 60 per time point. (Scale bar, 5 µm.) (*C* and *D*) Direct comparison of the temporal MFI of HRS and EPN1 at 2.5, 5, 15, and 30 min relative to the max intensity of each protein at 5 min (*C*), and the temporal PCC between HRS and TCRβ compared to the temporal PCC of EPN1 and TCRβ (*D*) from the micrographs in *A* and *B*. Lines are median ±SD.

We incubated mCD4 T cells on SLBs with ICAM-1 alone for 5 min or with ICAM-1 plus I-E^k^-MCC for 2.5, 5, 15, and 30 min before we fixed and permeabilized them. We then immunolabeled HRS and observed potent recruitment in response to antigen already after 2.5 min with a twofold increase in mean fluorescence intensity (MFI) compared to no antigen (Fig. 2*A*). As for clathrin, HRS recruitment peaked after 5 min before the fluorescence intensity dropped after 15 min (*SI Appendix*, Fig. S2*A*). The early recruitment correlated with extensive colocalization with TCR microclusters with median PCCs of 0.80 and 0.77 at 2.5 and 5 min, respectively. This was reduced to 0.49 at 15 min and 0.45 at 30 min (*SI Appendix*, Fig. S2*B*).

We have also previously reported that the endocytic clathrin adaptor EPN1 localizes to the IS during T cell activation (35). Here, we confirm those observations and show that as for HRS and CHC, this recruitment depends on TCR–pMHC ligation (Fig. 2*B*). However, while HRS and CHC recruitment peaked after 5 min, EPN1 recruitment peaked after 15 min with a threefold increase in MFI compared to no antigen (*SI Appendix*, Fig. S2*C*). EPN1 colocalized with TCR with a PCC of 0.38 at 2.5 min, 0.52 at 5 min, 0.51 at 15 min, and 0.44 at 30 min (*SI Appendix*, Fig. S2*D*). When directly compared to HRS, we observed significantly less EPN1 recruitment at 2.5 min and 5 min relative to the maximum MFI at 5 min. However, after 15 and 30 min, this was reversed with significantly more EPN1 recruited than HRS (Fig. 2*C*). In terms of colocalization with TCR, this was significantly higher for HRS after 2.5 and 5 min but at the same level for HRS and EPN1 after 15 and 30 min (Fig. 2*D*).

**Fig. 3.**
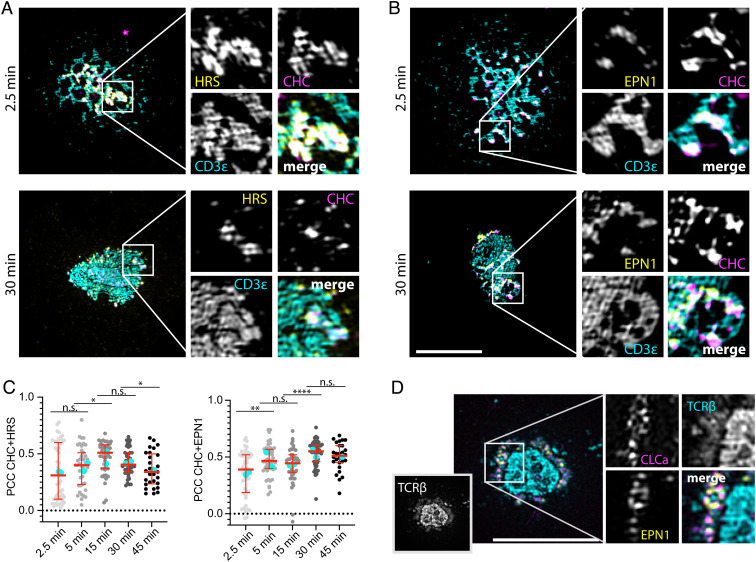
HRS and EPN1 colocalize with clathrin at TCR microclusters. (*A* and *B*) Representative TIRF–SIM micrographs of hCD4 T cells incubated on SLBs with ICAM-1-AF405 (200/µm^2^) + anti-CD3ε UCHT1-AF488 (cyan, 30/µm^2^) for the indicated times and labeled with anti-CHC (magenta) and anti-HRS (*A*, yellow) or anti-EPN1 (*B*, yellow). (*C*) Quantification of the temporal PCC between CHC and HRS or EPN1 across the synaptic interface. N_cells_ ≥ 26. (Scale bar, 5 µm.) Lines are median value ± IQR, and cyan dots are average values from individual experiments. (*D*) Representative TIRF–SIM micrograph of an AND mCD4 T cell expressing CLCa-mCherry (magenta) incubated on SLB with ICAM-1-AF405 (200/µm^2^) + I-E^k^-MCC (20/µm^2^) for 20 min, fixed, permeabilized, and labeled with anti-TCRβ (cyan) and anti-EPN1 (yellow). (Scale bar, 5 µm.)

We next applied TIRF–SIM to investigate the temporal overlap between CHC and HRS or EPN1 in hCD4 T cells as before. We then observed that both HRS and EPN1 were recruited to the coalescing TCR microclusters and that they overlapped extensively with CHC in these areas within 2.5 min of activation (Fig. 3 *A* and *B*). However, while HRS and CHC colocalization peaked at 15 min, EPN1 and CHC colocalization plateaued and remained high even after 45 min at the periphery of the cSMAC (Fig. 3*C*). We then examined the overlap between CLCa and EPN1 in mCD4 T cells after 20 min of activation with pMHC followed by TCRβ labeling with the H57 Fab. In contrast to the hCD4 T cells labeled with anti-CD3ε attached to the SLB, the H57 Fab–labeled global TCRβ. This enabled us to distinguish the TCRβ in extracellular vesicles from the TCRβ in the plasma membrane, and we observed that EPN1 and CLCa primarily localized to the plasma membrane fraction (Fig. 3*D*). Note that there is a visible cleft between the TCRβ in the central region of the cSMAC and the TCRβ in the plasma membrane.

STAM2 is known to form the ESCRT-0 complex together with HRS (20). We therefore investigated whether this protein is also recruited to the IS during AND mCD4 T cell activation and observed a similar recruitment pattern to HRS with peak recruitment after 5 min followed by a strong reduction in fluorescence intensity after 20 min (*SI Appendix*, Fig. S2 *E* and *F*). This was accompanied by a high degree of colocalization between STAM2 and CHC or TCRβ at 5 min with PCCs of 0.62 and 0.64, respectively, which dropped to 0.40 and 0.31 after 20 min (*SI Appendix*, Fig. S2 *G* and *H*).

AP2 is regarded as the primary endocytic clathrin adaptor and is an integral part of the majority of clathrin-coated pits (23). We therefore analyzed temporal AP2 recruitment to the IS as well and observed a significant increase in response to antigen stimulation (*SI Appendix*, Fig. S2 *I* and *J*). However, AP2 did not colocalize with TCR microclusters (*SI Appendix*, Fig. S2*K*).

Taken together, these results show that clathrin adaptor recruitment to the IS is regulated in a temporal manner initially being dominated by the ESCRT-0 components HRS and STAM2 and subsequently by the endocytic adaptor protein EPN1. AP2 is recruited to the plasma membrane in response to T cell activation but not to TCR microclusters.

### Clathrin, HRS, STAM2, and TSG101 Are Required for cSMAC Formation.

**Fig. 4.**
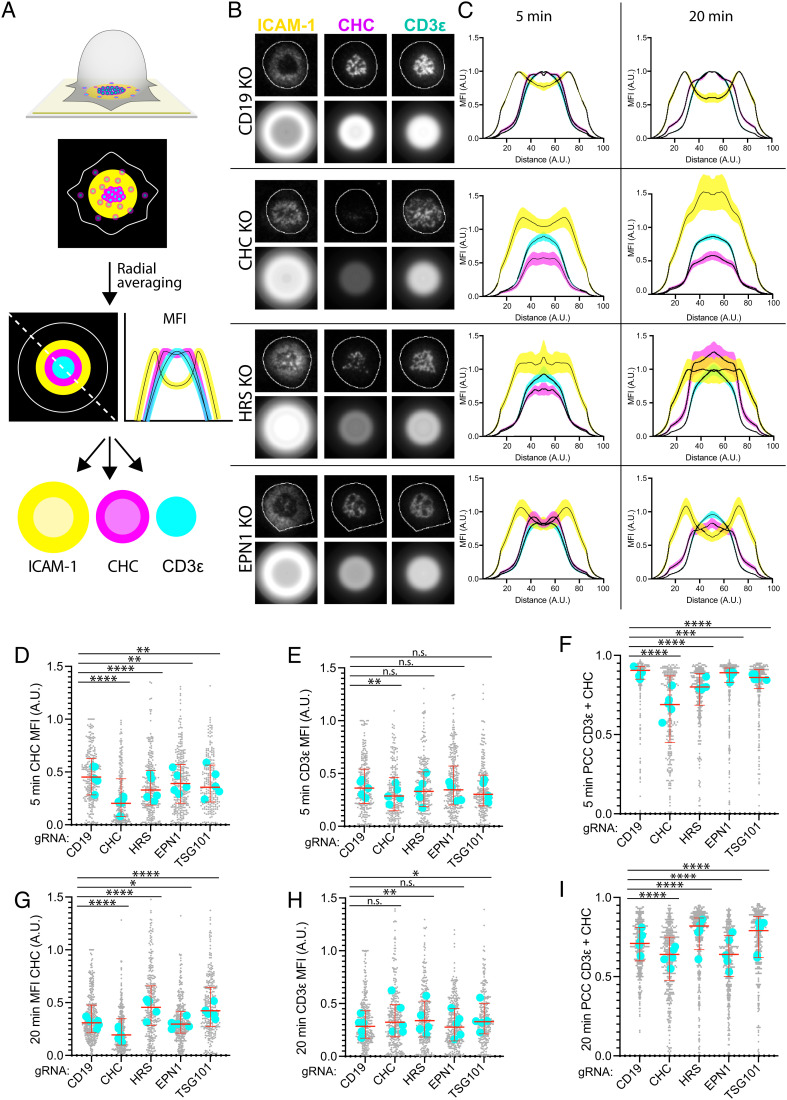
HRS and EPN1 recruit clathrin to the IS in a sequential manner. (*A*) Schematic of radial averaging of micrographs of the IS formed between a T cell and an SLB. ICAM-1 is labeled yellow, TCR cyan, and CHC magenta. The dashed line and the line plots represent a diagonal measurement of the positional MFI of the individual channels. (*B*) Representative TIRF micrographs and corresponding radial averages of hCD4 CD19, CHC, HRS, and EPN1 KO T cells incubated on SLBs with ICAM-1-AF405 (200/µm^2^) and anti-CD3ε UCHT1-AF488 (30/µm^2^) for 5 min. (*C*) Radial averages of hCD4 CD19, CHC, HRS, and EPN1 KO T cells incubated on the SLBs from *B* for 5 and 20 min. The MFI represents MFI from five individual experiments ± SEM. (*D*–*I*) Quantification of the MFI of CHC (*D* and *G*), CD3ε (*E* and *H*), and the PCC between CHC and CD3ε (*F* and *I*) across the synaptic interface from hCD4 CD19, CHC, HRS, EPN1, and TSG101 KO T cells incubated on the SLBs from *B* for 5 and 20 min. N_cells_ ≥ 219 per condition. Lines are median ± IQR, and cyan dots are median values from individual experiments.

We next depleted CHC, HRS, EPN1, AP2, and STAM2 by CRISPR/Cas9-mediated knockout (KO) in hCD4 T cells and analyzed IS formation on SLBs with UCHT1-AF488 (30 µm^−2^) and ICAM-1-AF405 (200 µm^−2^) after 5 and 20 min. CD19 and TSG101 KO were included as negative and positive controls, respectively, and KO efficiency was determined by immunoblotting (*SI Appendix*, Fig. S3*A*). To analyze the synaptic distribution of ICAM-1, CHC, and UCHT1 in response to the KOs, we segmented individual cells and performed radial averaging on the segmented micrographs (Fig. 4 *A* and *B*). We observed defective cSMAC formation following clathrin, HRS, STAM2, and TSG101 KO, with UCHT1-labeled CD3ε appearing unable to fully translocate to the center of the contact area and ICAM-1 being incompletely excluded from the cSMAC after 20 min as reported following siRNA-mediated TSG101 knockdown previously (15) (Fig. 4*C* and *SI Appendix*, Fig. S3*B*). For HRS and TSG101 KO, this was accompanied by a strong increase in cSMAC localized CHC, while for CHC KO, the ICAM-1 ring seemed to collapse into the cSMAC.

When we then examined clathrin recruitment to the synaptic interface in these cells, we observed a significant drop following HRS, EPN1, STAM2, and TSG101 KO after 5 min of incubation on SLBs, thus confirming that these proteins are indeed recruiting clathrin there at this time point (Fig. 4*D* and *SI Appendix*, Fig. S3*C*). We also detected a drop in CD3ε MFI after 5 min following CHC KO which might indicate reduced TCR expression at the plasma membrane, possibly due to defective TCR recycling (Fig. 4*E*). When we analyzed colocalization between CHC and CD3ε after 5 min, we observed a strong decrease following CHC and HRS KO compared to the other conditions (Fig. 4*F* and *SI Appendix*, Fig. S3*D*). While there was also a significant decrease following EPN1, STAM2, and TSG101 KO, these data suggest that HRS is the primary adaptor protein for recruiting clathrin to TCR microclusters early during IS formation.

Intriguingly, after 20 min of incubation on the SLBs, clathrin recruitment was strongly increased following HRS and TSG101 KO, while we observed a slight decrease following EPN1 and STAM2 KO (Fig. 4*G* and *SI Appendix*, Fig. S3*E*). We observed a slight increase in CD3ε MFI following AP2, HRS, and TSG101 KO as reported previously for TCRβ following siRNA knockdown of HRS and TSG101 in AND T cells (15) (Fig. 4*H* and *SI Appendix*, Fig. S3*F*). When we examined colocalization between CHC and UCHT1, we observed a strong decrease following CHC and EPN1 KO, while there was now a strong increase following HRS KO and TSG101 KO (Fig. 4*I*). STAM2 KO also led to a slight decrease (*SI Appendix,* Fig. S3*G*).

These data suggest that EPN1 is the primary adaptor protein for recruiting clathrin to the mature cSMAC, while HRS and TSG101 KO seem to induce defective clearance of activated TCR from the plasma membrane and clathrin arrest. Interestingly, STAM2 KO seems to decrease clathrin recruitment both early and late during IS formation, thus indicating that it not only acts as a partner for HRS in the ESCRT-0 complex in this context but also has an additional role during late-stage IS formation. AP2 KO did not affect clathrin recruitment at any time point, but the slight increase in CD3ε MFI after 20 min might imply that TCR/CD3 surface expression had increased due to diminished steady state turnover as this has been reported to be mediated by AP2 and clathrin previously (45).

We next investigated clathrin and CD3ε in the mature cSMAC following CD19, CHC, and HRS KO by TIRF–SIM (*SI Appendix*, Fig. S3*H*). We then clearly observed CHC at the edge of the cSMAC after 20 min following CD19 KO. However, while for the CD19 KO cells the CD3ε pattern appeared highly punctate, it appeared as an interconnected web-like structure in the CHC and HRS KO cells similar to the coalescing TCR domains observed after 5 min in *SI Appendix*, Fig. S1*B*. The consolidated central UCHT1 engagement observed following CD19 KO might thus represent released TCR loaded synaptic ectosomes, while the poorly centralized web-like and lobular patterns of UCHT1 engagement that dominated following CHC and HRS KO might represent engaged CD3/TCR confined in the interface between the plasma membrane and the SLB.

### HRS and Clathrin Are Required for Transfer of TCR Loaded Vesicles.

**Fig. 5.**
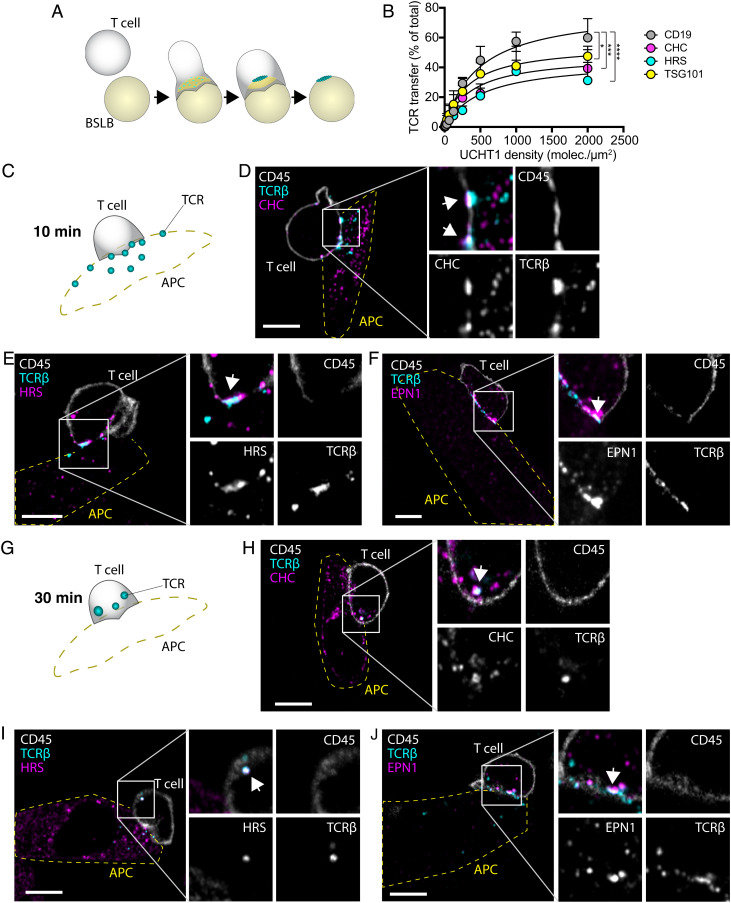
Clathrin and HRS are required for transfer of TCR loaded vesicles. (*A*) Schematic of vesicular transfer from T cells to BSLBs with ICAM-1 (yellow) and UCHT1 (cyan). As the TCR microclusters reach the center of the contact area, they are released as synaptic ectosomes which are left behind as the T cell detaches from the bead. (*B*) Quantification of the fluorescence intensity of beads with immunolabeled TCR transferred from CD19, CHC, HRS, and TSG101 KO hCD4 T cells at increasing UCHT1 densities. The dots represent MFI ± SEM of the beads relative to the sum MFI of the beads and the cells from three experiments. The *P* values were calculated with the F test. (*C*–*J*) Illustrations and micrographs of slices from Airyscan^®^ Z-stacks with a step size of 250 nm of mCD4 AND T cells incubated with CHO-I-E^k^ APCs for 10 min (*C*–*F*) and 30 min (*G*–*J*) and immunolabeled with anti-CD45, anti-TCRβ, and anti-CHC, anti-HRS, or anti-EPN1. The outline of the APCs is indicated with a yellow dashed line. White arrows indicate overlap between TCRβ and CHC, HRS, or EPN1. (Scale bar, 5 µm.)

To examine whether these proteins are involved in release of TCR loaded vesicles, we next investigated TCR transfer to bead supported lipid bilayers (BSLBs) as illustrated in Fig. 5*A*. This was done by incubating human CD4 T cells with SLB-coated silica beads at a 1:1 ratio for 90 min before detaching the BSLB and analyzing them by flow cytometry [described in Saliba *et al.* (35)]. We observed a strong reduction in the amount of TCR transferred to the beads following CHC and HRS KO, calculated as the amount of TCR transferred to the beads normalized to the total amount of TCR on the cells and the beads (Fig. 5*B*). These data demonstrate that clathrin and HRS are essential for transfer of TCR at the IS.

To confirm that clathrin, HRS, and EPN1 recruitment to the IS also occurs between T cells and APCs, we incubated CHO cells expressing the I-E^k^-MCC pMHC complex (CHO-I-E^k^-MCC) with AND mCD4 T cells for 10 and 30 min before we fixed and permeabilized them. We then immunolabeled the cells with antibodies targeting each of the proteins together with H57 Fab to label TCRβ and anti-CD45 to label the T cells. Airyscan® microscopy of the labeled cells revealed that clathrin, HRS, and EPN1 were all recruited to the synaptic interface and colocalized with TCRβ after 10 min (Fig. 5 *C*–*F*). Note that the T cells had already released TCR onto the APCs at this time point and that CD45 is excluded from TCR release sites.

After 30 min, most of the TCR was either transferred to the APCs or internalized by the T cell (Fig. 5 *G*–*J*). This was accompanied by a remarkable shift in localization of HRS and clathrin on the T cell side, a large fraction of which is now associated with the TCR loaded internalized vesicles likely representing early/late endosomes. EPN1 on the other hand still appeared to associate with TCR at the plasma membrane.

When we also directly immunolabeled I-E^k^-MCC, we observed that the internalized TCR colocalized extensively with the pMHC complex, thus indicating that the endosomal structures represent cointernalized antigen–TCR conjugates (*SI Appendix*, Fig. S4*A*). Some of these structures appeared to originate from microvillar protrusions from the APC interacting with the T cell body distal from the T cell–APC interface which colocalized with CHC staining on the TCR side (*SI Appendix*, Fig. S4*B*). Similar protrusions originating from the T cell also reached distant APCs and other T cells, as previously reported by Kim *et al.* (8). As shown in *SI Appendix*, Fig. S4 *C* and *D*, HRS and CHC could be found at the base of such structures, thus suggesting that these proteins might also regulate this form of membrane transfer. *SI Appendix*, Fig. S4*C*, shows how an individual AND T cell can form four different types of interactions with APCs and other T cells simultaneously, all of which are marked by HRS on the T cell side: 1. primary IS, 2. secondary IS, 3. microvillar protrusion from the APC, and 4. microvillar protrusion from the T cell.

**Fig. 6.**
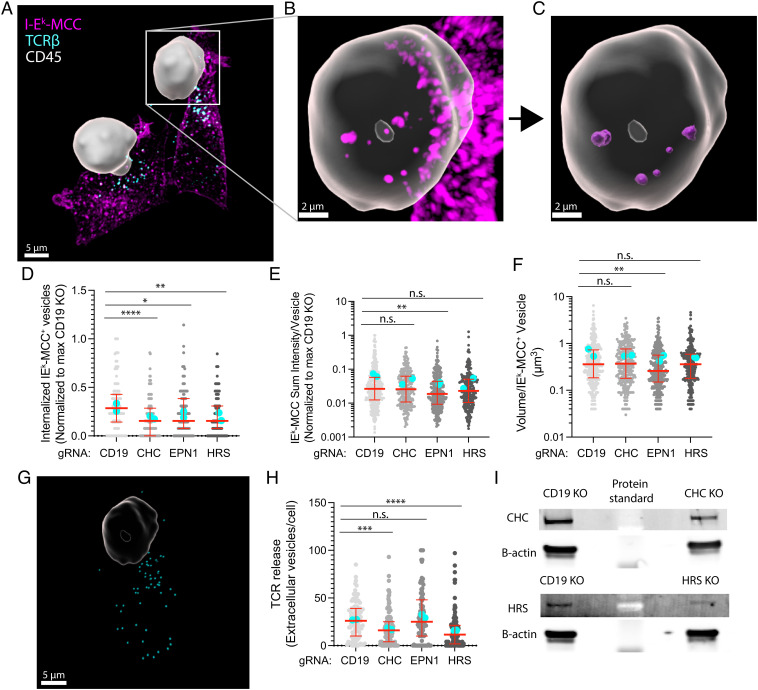
Clathrin, HRS, and EPN1 regulate pMHC–TCR trans-endocytosis. (*A*–*C*) Imaris 3D reconstructed micrographs of spinning disc confocal Z-stacks with a step size of 250 nm of mCD4 AND T cells incubated with CHO-I-E^k^ APCs for 30 min and immunolabeled with anti-CD45 (white), anti-TCRβ (cyan), and anti-I-E^k^-MCC (magenta). The CD45 signal has been used to create a mask of the T cell. (*D*–*F*) Quantification of the number of internalized I-E^k^-MCC–positive vesicles per cell (*D*), sum I-E^k^-MCC fluorescence intensity per vesicle (*E*), and volume per I-E^k^-MCC–positive vesicle (*F*) following KO of CD19, CHC, EPN1, or HRS in mCD4 AND cells incubated for 30 min on CHO-I-E^k^ APCs. N_cells_ ≥ 111 per condition. Lines are median value ± IQR, and cyan dots are average values from individual experiments. (*G*) Segmented TCR-positive vesicles (cyan) released from one of the mCD4 T cells from (*A*). (*H*) Quantification of the number of released TCR-positive vesicles per cell. (*I*) A representative WB of the protein levels of CHC, HRS, and β-actin from one experiment following CHC and HRS KO.

We then performed CRISPR/Cas9-mediated KO of CD19, CHC, HRS, and EPN1 in the AND cells and incubated them with CHO-I-E^k^ cells for 30 min before we fixed and permeabilized them as before. We immunolabeled TCRβ, I-E^k^-MCC, and CD45 and used Imaris^®^ software to create a 3D mask of the T cells based on the CD45 signal (Fig. 6*A*). This enabled us to segment I-E^k^-MCC–positive vesicles internalized by the T cells (Fig. 6 *B* and *C*). When we counted the number of such vesicles per T cell, we observed a median decrease of 50% following KO of CHC, HRS, and EPN1 (Fig. 6*D*). When we then quantified the sum intensity of I-E^k^-MCC per vesicle, we observed a significant reduction following EPN1 KO (Fig. 6*E*). The drop in intensity was apparently caused by a reduction in the volume of the internalized vesicles as this parameter was also affected by EPN1 KO but not by depletion of CHC or HRS (Fig. 6*F*). We then segmented extracellular vesicles released by the T cells based on their H57 signal, disregarding any signal originating from within the T cell body (Fig. 6*G*). When we counted the number of released vesicles per T cell, we observed a strong reduction following CHC and HRS KO but not after EPN1 depletion (Fig. 6*H*). Fig. 6*I* shows a representative western blot (WB) of the protein levels of CHC and HRS following KO.

We next examined TCRβ accumulation in the cSMAC of AND cells activated on SLBs for 5 and 20 min following KO of EPN1 and HRS. While there was no significant difference in TCRβ MFI after 5 min, we could observe an increase following EPN1 KO and a decrease following HRS KO after 20 min (*SI Appendix*, Fig. S5 *A* and *B*). This suggests that EPN1 is required to remove TCR from the cSMAC, while HRS is required to accumulate it there. When we analyzed the corresponding MFI of EPN1 and HRS, we observed efficient depletion of both proteins following KO (*SI Appendix*, Fig. S5 *C* and *D*).

Taken together, these data show that CHC, EPN1, and HRS are all involved in pMHC–TCR internalization on the T cell side, while only CHC and HRS are involved in release of TCR-positive extracellular vesicles.

## Discussion

We have demonstrated that clathrin orchestrates two alternative fates for triggered TCR in primary T cells activated by cognate pMHC. First, we show that clathrin is recruited to TCR microclusters by HRS within 2.5 min of T cell activation where it mediates budding of TCR loaded synaptic ectosomes from the plasma membrane into the synaptic cleft. Depletion of CHC and HRS, but not EPN1, blocks this process. Then, about 2.5 min later, EPN1 initiates clathrin-mediated internalization of TCR conjugated to pMHC. Depletion of EPN1, HRS, or clathrin blocks pMHC–TCR endocytosis. The decrease in antigen uptake following HRS KO might be caused by the block in TCR release and CHC arrest at the cSMAC as we show after HRS and TSG101 KO in human CD4 T cells activated on SLBs. However, we cannot exclude the possibility of HRS being directly involved in pMHC–TCR endocytosis. AP2 is present at the synaptic interface but is not specifically recruited to TCR microclusters and is not required for release or internalization of triggered TCR.

Several studies have indicated that clathrin is not involved in uptake of triggered TCR (30, 33, 34). However, the conclusions regarding clathrin in those studies have been based on work in the Jurkat cell line, which may not faithfully represent receptor trafficking in primary T cells. They have also relied on indirect analysis of CD3 expression on the cell surface or on quantification of internalized TCR-positive vesicles near the IS and not direct analysis of total TCR- and pMHC-positive vesicles within the T cells. We show that antigen can be internalized from the tips of microvillar protrusions extending from the APC, far from the T cell–APC interface. Furthermore, some of those studies have been conducted in systems where T cells have been activated on inert substrates (i.e., antigen-coated plastic surfaces) from which the T cells might not be able to internalize antigen–TCR conjugates. Indeed, we also fail to detect vast uptake of TCR conjugated to UCHT1 from SLBs perhaps due to the combination of high affinity of the antibody for CD3 and the flatness and/or stiffness of the SLB (46). Direct TCR triggering might also be required for initiation of endocytosis as we observe increased TCRβ accumulation in the cSMAC following EPN1 KO in AND T cells activated on SLB with cognate pMHC but not in hCD4 cells activated on SLB with the anti-CD3ε UCHT1-Fab. We also observe clear defects in pMHC–TCR uptake after CHC, EPN1, and HRS KO when directly counting the number of antigen-positive vesicles internalized by primary T cells following activation on APCs.

We show that HRS and EPN1 operate sequentially with an offset of a few minutes. EPN1 has been reported to associate with polyubiquitinated receptors but has low affinity for monoubiquitinated receptors (47). HRS on the other hand has been shown to associate stably with monoubiquitinated cargos (48). Ubiquitin ligases are recruited to TCR microclusters in a specific temporal sequence, and the CD3 chains of the TCR complex undergo direct ubiquitination (49, 50). A requirement of CD3 polyubiquitination for EPN1 recruitment compared to sufficiency of monoubiquitination for HRS recruitment could thus explain the sequential TCR microcluster association of these clathrin adaptors.

We demonstrate that TCR loaded vesicles are rapidly released directly from the plasma membrane upon T cell activation in a process mediated by clathrin and the ESCRT machinery. There is no specific term for the process described here of clathrin and ESCRT-dependent evagination of the plasma membrane into small extracellular vesicles. Formation of large vesicles at the plasma membrane can take place through a process of blebbing (51). Given the precedent of referring to small vesicles that bud from the plasma membrane as ectosomes (52), we suggest clathrin- and ESCRT-mediated ectocytosis (CEME) as a term to describe this process.

Previous reports have shown that bystander TCR can be internalized from the plasma membrane and recycled to the IS (33, 53). We suspect that part of this TCR fraction might be incorporated into ILVs in multivesicular bodies and released at the IS by exocytosis and that this process also involves clathrin and the ESCRT machinery. This is supported by the finding that HRS- and clathrin-positive vesicles polarize to the IS during T cell activation (21). However, this process appears to be EPN1 independent as we did not observe any reduction in TCR transfer following EPN1 KO. In contrast, EPN1 KO has been shown to reduce transfer of CD40 ligand potentially by preventing its uptake upstream of ILV formation (35).

Following a temporal shift in clathrin adaptor recruitment, EPN1- and clathrin-mediated internalization of antigen-ligated TCR is initiated. The active capture of ligands from one cell by another has been referred to as trans-endocytosis or trogocytosis (54–56). These terms primarily differ with regard to the posttransfer fate of the captured ligands, trans-endocytosis indicating degradation, and trogocytosis indicating surface expression. While we cannot exclude the possibility of captured pMHC being expressed by the T cells in our system, most of the ligands appear to accumulate in late endosomes. We therefore refer to this process as clathrin-mediated trans-endocytosis.

In conclusion, the data presented in this manuscript establish clathrin, EPN1, and the ESCRT machinery as essential players in regulating bidirectional membrane transfer between T cells and APCs.

## Materials and Methods

UCHT1 anti-CD3ε Fab was prepared from UCHT1 IgG (Bio X Cell, Lebanon, NH) by pepsin digestion followed by gel filtration, reduction in the hinge disulfides, and reaction with maleimide-PEG2-biotin (Thermo Fisher Scientific, #21901BID). These were then labeled with NHS esters of fluorescent dyes at ~1 fluorophore per Fab ratio. Recombinant ICAM-1-12His was generated in S9 cells, purified by Ni2+ affinity, and labeled with Alexa Fluor 405–NHS ester. Anti-mouse TCRβ clone H57-597 Fab (Bio X Cell, Lebanon, NH) was prepared from anti-mouse TCRβ clone H57-597 (BioLegend, #109201) as before. Primary antibodies are rabbit anti-EPN1 (Abcam, #ab75879), rabbit anti-CHC (Abcam, #ab21679), rabbit anti-HRS (Abcam, #72053), rabbit anti-TSG101 (Abcam, #125011), rabbit anti-AP2M1 (Thermo Fisher Scientific; #MA5-32360), rabbit anti-STAM2 (Abcam, #151545), mouse CD45 Alexa Fluor^®^ 405–conjugated antibody (Bio-Techne, #FAB114V), mouse I-E^k^/rat RT1D Alexa Fluor^®^ 647–conjugated antibody (BioLegend, #110211), mouse anti-β-actin (Merck Life Science, #A5316), and Phalloidin-647 (Thermo Fisher Scientific, #A22287). Secondary antibodies are Anti-rabbit Alexa Fluor^®^ 568 (Molecular Probes, #A10042), donkey anti-mouse IRDye 680 (LI-COR Biosciences, #926-32222), and donkey anti-rabbit IRDye 800 (LI-COR Biosciences, #926-32213).

### Planar Supported Lipid Bilayer (PSLB) Experiments.

A total of 1 × 10^5^ T cells were added to each PSLB channel (*SI Appendix*, *Materials and Methods*) and incubated at 37 **°**C for the indicated times. Cells were fixed with 100 μL of 4% paraformaldehyde (PFA) in PHEM buffer (60 mM PIPES, 25 mM HEPES, 10 mM EGTA, and 4 mM MgSO_4_·7H_2_O) for 10 min, washed, and permeabilized with 100 μL of 0.1% Triton X-100 in 0.1% BSA/HBS for 2 min. The channels were washed three times with 200 μL PBS before blocking solution with 5% goat serum or BSA in PBS was added for 1 h. Antibodies were then diluted in 200 μL blocking solution and incubated with the cells overnight at 4 °C. Each channel was then washed three times with 200 µL PBS before the appropriate secondary antibody was added and incubated for 1 h. The channels were washed again three times with 200 µL PBS.

TIRFM imaging was performed either with an Olympus IX83 inverted microscope (Keymed, Southend-on-Sea, UK) equipped with a 150× 1.45 NA oil immersion objective and an EMCCD camera (Evolve Delta, Photometrics, Tucson, AZ) or a DeltaVision OMX V4 System (Applied Precision, GE Healthcare) equipped with a 60× ApoN NA 1.49 objective (Olympus) and three cooled sCMOS cameras (PCO). For live-cell imaging, the stage and objective were temperature controlled at 37 °C, and the sample holder was enclosed by a humidified 5% CO_2_ incubation chamber.

eTIRF–SIM imaging was performed with a custom-built eTIRF–SIM setup described in detail elsewhere (57, 58). The excitation angle was adjusted to ensure <200 nm penetration depth with respect to the basal plane. Images were then reconstructed with a custom algorithm and chromatic aberration corrected with MultiStackReg Fiji plugin (https://biii.eu/multistackreg). TetraSpeck beads of 100 nm were used as a reference sample.

### CRISPR/Cas9-Mediated KO.

Freshly isolated CD4^+^ T cells were activated for 3 d on CD3/CD28 activation beads (Thermo Fisher Scientific, Loughborough, UK; #11132D) as before. They were then washed three times in Opti-MEM (Gibco; #11058021). Then, 1 μL 200 μM crisprRNA was mixed with 1 μL of 200 μM tracrRNA in an RNase-free PCR tube and incubated in a C1000 Thermal Cycler (Bio-Rad) at 95 °C for 5 min and allowed to cool to RT slowly. Subsequently, 7.5 µL of 20 µM Cas9 was added to the guide RNA complex at room temperature while swirling the pipette to mix the reagents. The mix was then incubated in the Thermal Cycler at 37 °C for 15 min and slowly cooled to room temperature. Finally, 1 μL of 200 μM electroporation enhancer was added to the RNP mix at RT and gently mixed. RNAs (*SI Appendix*, Table S1), electroporation enhancer, and Cas9 were from IDT DNA.

Several crisprRNAs were tested per target protein and analyzed by microscopy to determine which target sequence resulted in the strongest KO. This was confirmed by WB for all proteins except mouse EPN1 which we failed to detect despite testing multiple antibodies. However, by microscopy, we detected an EPN1 reduction of 70% following KO by Mm.Cas9.EPN1.1.AA (*SI Appendix*, Fig. S5).

### Data Analysis.

Automated segmentation of individual cells from each TIRF micrograph was performed with FIJI (version 2.3.0/1.53f) based on the bright-field channel, followed by background subtraction (rolling ball, 50 pixel radius). Calculation of relative fluorescence intensity was done using MATLAB (version 2021b). 3D segmentation and quantification of confocal Z-stacks were done using Imaris batch analysis (version 9.8.0). For radial averaging, the Fiji macro (https://github.com/donFellus/radAv) was used. This macro is specifically designed to analyze the average position of subcellular structures within the IS. It rotates a selected image 360 times 1° and creates a copy following each rotation. It then merges all the copies to create a radial positional average of the fluorescence signals in the original image.

TCR microclusters were tracked using the ImageJ plugin TrackMate (59), followed by MATLAB-based analysis (https://github.com/donFellus/loadFijiTracks). Statistical analysis and plots were generated using GraphPad Prism (version 9.2.0). Analysis of data generated by flow cytometry was performed with FlowJo LLC (version 10.8).

### Statistics.

Unless otherwise stated, lines and error bars indicate median value ± interquartile range (IQR); cyan/magenta/yellow dots indicate average measurements from individual experiments, and statistical analysis was done with the unpaired two-tailed nonparametric Mann–Whitney test. * = *P* value < 0.05, ** = *P* value < 0.01, *** = *P* value < 0.001, **** = *P* value < 0.0001.

## Supplementary Material

Appendix 01 (PDF)

Movie S1. **Clathrin is recruited to TCR microclusters**. Representative movie of an AND T cell expressing CLCa-mCherry (magenta) incubated on an SLB for 5 min with ICAM-1-AF405 (200/μm^2^) and I-E^k^-MCC (50/μm^2^). The TCR is labelled with anti-TCRβ (cyan). The framerate is 0.3 fps.

## Data Availability

All data shared in this article appears in the main text and *SI Appendix*. The raw data pertaining to the images in the manuscript has been uploaded to OSF: OSF.IO/SXEQR.
