# Supplementary material for: Clathrin mediates both internalization and vesicular release of triggered T cell receptor at the immunological synapse
Source: Proc Natl Acad Sci U S A. 2023 Feb 2;120(6):e2211368120. doi: 10.1073/pnas.2211368120 (PMC9963302; doi:10.1073/pnas.2211368120)
Supplement: Supplementary file 1 — Appendix 01 (PDF) [file pnas.2211368120.sapp.pdf]

Further methods

Cell culture

Human T cells: De-identified human peripheral blood samples were acquired from the National Health Service blood service under ethics license REC 11/H0711/7 (University of Oxford). $CD4^+$ T cells were isolated by negative selection (RosetteSepTM Human $CD4^+$ T cell Enrichment Cocktail, STEMCELL technologies, Cambridge, UK; #15023) following the manufacturer's protocol. Donor specific $CD4^+$ T cells were plated at $1x10^6$ cells/ml with CD3/CD28 activation beads (Thermo Fisher Scientific, Loughborough, UK; #11132D) at a 1:1 ratio with 50 U/ml IL-2 at 2 ml per well in a 24 well plate and expanded for three days at 37°C in a CO_2 incubator. On day three, cells were resuspended, counted and replenished with fresh media to reach the original density of $1x10^6$ cells/ml. The cells were expanded for four more days replenished with 50 U/ml IL-2 and fresh culture media every second day. The cells were left without IL-2 the day before they were incubated on SLBs.

Murine T cells: AND Mouse T cells were generated from spleen and lymph nodes of TcrAND B10.Br mice; mice were housed under pathogen-free conditions in the Kennedy Institute of Rheumatology Animal Facility in accordance with local and Home Office regulations. The mice were housed in individually ventilated cages (IVC) with corn cob bedding. 12 hours of light/dark cycle with half an hour of dim light period in place. Appropriate environmental enrichment was provided: Enviro-dri, and housing tunnel, cage balcony and chew blocks. The temperature was maintained at 21 degrees +/- 2 and the humidity 55% +/- 10. The protocols were reviewed by local Veterinary Surgeon (Vet) and Named Animal Care and Welfare Officer (NACWO) before being reviewed and approved by Animal Welfare and Ethical Review Body (AWERB). All procedures were conducted in accordance with the UK Scientific Procedures Act of 1986 and overlooked by University of Oxford Department of Biomedical Services, Clinical medicine ORC (old road campus).

AND T cells were plated at $1x10^7$ cells/ml with 1 µM MCC peptide with 50 U/ml IL-2 at 2 ml per well in a 24 well plate and expanded for three days at 37°C in a CO_2 incubator. On day three, cells were resuspended, counted and replenished with fresh media to reach a density of $1x10^6$ cells/ml. The cells were expanded for four more days replenished with 50 U/ml IL-2 and fresh culture media every second day. The cells were left without IL-2 the day before they were used in downstream procedures (AND T cell blasts).

Chinese hamster ovary (CHO) cells stably expressing I-E^k-MCC (CHO-I-E^k) were maintained by passage every 3 days in complete RPMI 1640 medium (ThermoFisher Scientific, Loughborough, UK; #31870074).

Preparation of planar supported lipid bilayers (PSLB)

Flow chambers (sticky-Slide VI 0.4, Ibidi, Thistle Scientific LTD, Glasgow, UK; #80608) were attached to cleanroom cleaned coverslips (SCHOTT UK Ldt, Stafford, UK; #1472315) and cured for thirty minutes. 50ul of 12.5 mol% DOGS-NTA(Ni) lipids in DOPC was loaded in each IBIDI channel and incubated for 20 minutes. All lipids were obtained from Avanti Polar Lipids (Alabaster, AL). The channels were washed three times with 200ul of 0.1% BSA/HBS (20 mM HEPES, 137 mM NaCl, 1.7 mM KCl, 0.7 mM Na_2PO_4, 5 mM glucose, 2 mM $MgCl_2$, 1 mM $CaCl_2$, pH 7.2) and blocked with 200ul of 2% BSA/HBS for 20 minutes. The channels were washed again three times with 200ul of 0.1% BSA/HBS before 200ul of protein mix (ICAM-1-AF405, UCHT1-AF488 or I-E^k-MCC) in 0.1% BSA/HBS was loaded and incubated for 20 minutes. The protein concentrations were calibrated by flow cytometry to yield the desired density (as described for BSLB below). The channels was washed again three times with 200ul of 0.1% BSA/HBS. Fluorescence Recovery After Photobleaching (FRAP) was then performed to control bilayer mobility on an inverted Olympus FV1200 confocal microscope. All incubation steps were done at room temperature.

Preparation of bead supported lipid bilayers (BSLB)

$1x10^6$ non-functionalized silica beads (5.0 +/- 0.05 µm diameter, Bangs Laboratories, Inc.) per condition were washed three times with PBS in 1.5 ml conical microcentrifuge tubes. Then, BSLBs were formed by incubating washed silica beads with 12.5 mol% DOGS-NTA(Ni) DOPC. All liposome stocks mixed for the formation of SLB were used at a final lipid concentration of 0.4 mM. Then, to

remove excess lipids, the freshly formed BSLB were washed three times with 1% HSA/HBS. BSLBs were then blocked with 5% BSA, 100 µM NiSO4 in PBS for no longer than 20 minutes. Protein dilutions for BSLBs with UCHT1, ICAM-1 and CD40 were calibrated by making serial 2x dilutions of the desired protein from 1/100 to 1/50200. 100µl of each dilution was added to the beads and incubated for 20 minutes on a shaker at 800 rpm before the beads were washed three times in 1000ul of 0.1% BSA/HBS and analyzed by flow cytometry. Standard calibration curves were calculated using Quantum Alexa Fluor 647 MESF (Bangs Laboratories, Fishers, IN; # 647-A) or Quantum Alexa Fluor 488 MESF (Bangs Laboratories, Fishers, IN; #488-A).

Prior to incubation with BSLBs, T cells were washed two times with fully supplemented Phenol Red-free RPMI 1640 lacking IL-2 and resuspended to an assay concentration of 2.5×10^6 cells/mL. Then, T cells (2.5×10^5/well) were incubated with BSLB at 1:1 ratio in U-bottom 96 well plates (Corning) for 90 min at 37°C and 5% CO2. For gentle dissociation of BSLB:cell conjugates, culture plates were gradually cooled down by incubation at RT for 15 min, followed by incubation on ice for a minimum of 40 min. Then, cells and BSLB were centrifuged at 500 x g for 5 min and then gently resuspended in ice-cold 5% BSA in PBS prior to staining for flow cytometry analysis.

T cell activation on CHO-I-E^k cells

1×10^4 CHO-I-E^k cells were plated in each channel of 6 channel flow chambers (µ-Slide VI 0.4, Ibidi, Thistle Scientific LTD, Glasgow, UK; # 80606) for 24h in complete RPMI 1640 medium (Thermo Fisher Scientific, Loughborough, UK; #31870074) before 1×10^4 AND T cell blasts were added for 10 or 30 minutes. These were subsequently fixed with 100ul of 4% PFA in PHEM buffer (60mM PIPES, 25mM HEPES, 10mM EGTA, and 4mM MgSO4·7H20) for 10 minutes, washed and permeabilised with 100ul of 0.1% Triton X-100 in PBS for 2 minutes. The channels were washed three times with 200ul PBS before blocking solution with 5% BSA in PBS was added for one hour. Unconjugated primary antibodies were then diluted in 200 µl blocking solution and incubated with the cells overnight at 4°C. Each channel was then washed 3 times with 200 µl PBS before the appropriate secondary antibody was added and incubated for 1 hr. The channels were washed again 3 times with 200 µl PBS before Z-stacks were aquired either with a Zeiss AiryScan 880 confocal microscope or a Nikon Ti2-E microscope with a spinning disc.

Measurement of Synaptic Transfer by Flow Cytometry

Staining with fluorescent dye conjugated antibodies was performed immediately after dissociation of cells and BSLB conjugates. Staining was performed in ice-cold 5% BSA in PBS pH 7.4 (0.22 µm-filtered) for a minimum of 30 min at 4°C and agitation to avoid BSLB sedimentation (700 rpm in the dark). Then, cells and BSLB were washed three times and acquired immediately using an LSR Fortessa X-20 flow cytometer equipped with a High Throughput Sampler (HTS). For absolute quantification, we MESF beads which were first acquired to set photomultiplier voltages to position all the calibration peaks within an optimal arbitrary fluorescence units' dynamic range (between 10^1 and 2×10^5, and before compensation). Fluorescence spectral overlap compensation was then performed using single colour-labelled cells and BSLB, and unlabelled BSLB and cells. For markers displaying low surface expression levels unstained and single colour stained UltraComp eBeads (Thermo Fisher Scientific, #01-2222-42) were used for the calculation of compensation matrixes. After application of the resulting compensation matrix, experimental specimens and Quantum MESF beads were acquired using the same instrument settings. In most experiments acquisition was set up such as a minimum of 2×10^4 individual BSLB were recorded. To reduce the time of acquisition of high throughput experiments a minimum of 1×10^4 single BSLB was acquired per condition instead.

Data availability statement

The raw data pertaining to the images in the manuscript has been uploaded to OSF: OSF.IO/SXEQR

Table S1. Guide RNAs

Name	Sequence	Species
Mm.Cas9.CD19.1.AA	GGTGGAATGCTTCAGACGTC	Mouse
Mm.Cas9.CLTC.1.AC	CTAGCTGCATGCCCTTCAAT	Mouse
Mm.Cas9.HGS.1.AB	CCCCAAGTTCGGCATTGAGA	Mouse
Mm.Cas9.EPN1.1.AA	TGCCATCACGGTCCACATAC	Mouse
Hs.Cas9.CD19.1.AA	CTAGGTCCGAAACATTCCAC	Human
Hs.Cas9.CLTC.1.AA	CTGTACGGTAATTGATAATC	Human
Hs.Cas9.AP2M1.1.AA	ACGTTAAGCGGTCCAACATT	Human
Hs.Cas9.HGS.1.AA	GGGCATACAAGGCGACGTGT	Human
Hs.Cas9.EPN1.1.AA	CGAGAAGGCGACAACGTTGT	Human
Hs.Cas9.STAM2.1.AA	CAACCCCTTCGAGCAAGACG	Human
Hs.Cas9.TSG101.1.AA	CTATCCGCCATACCAGGCAA	Human

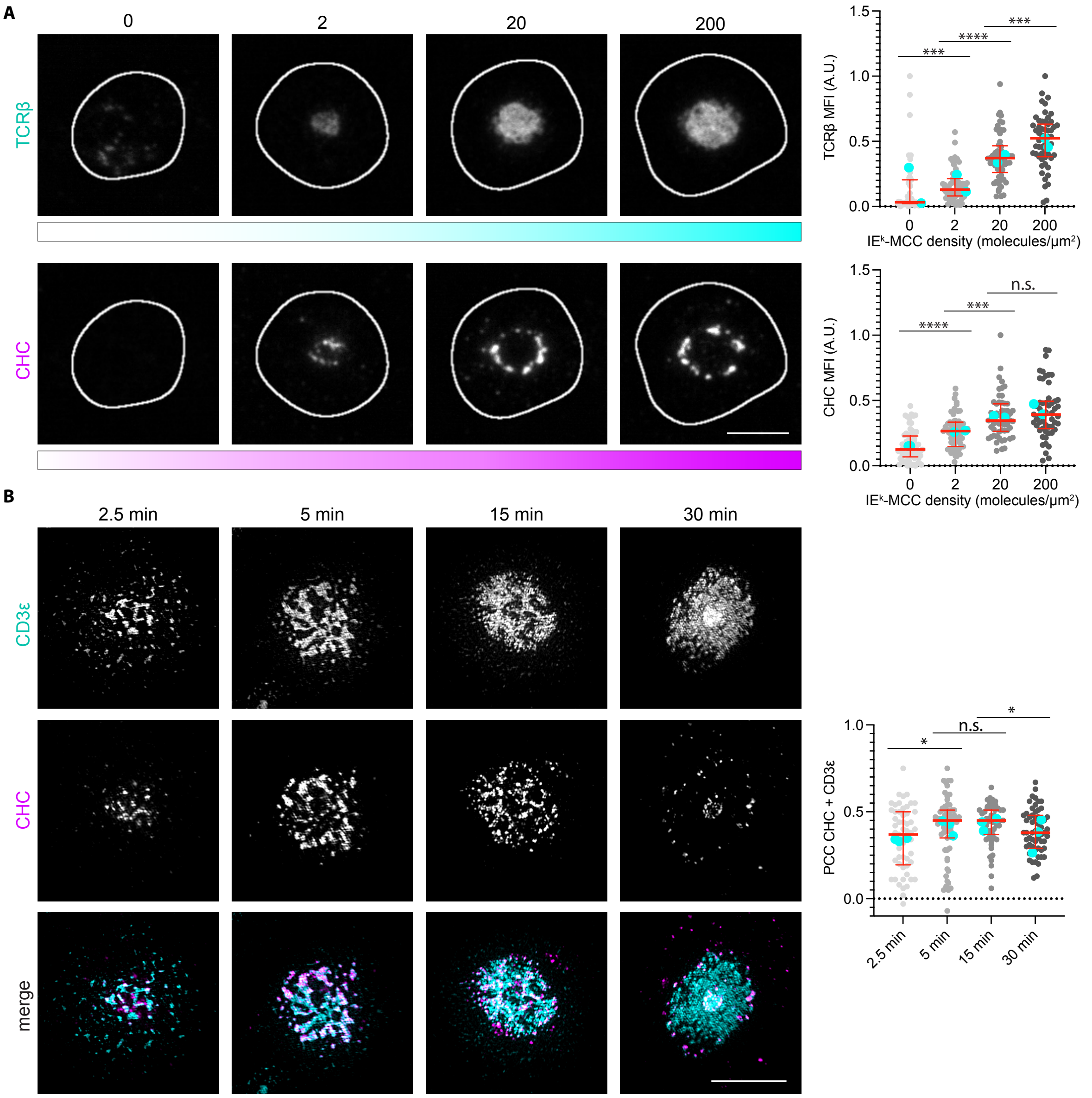

Fig. S1. Clathrin recruitment to the IS correlates with pMHC density. A Representative TIRF micrographs of AND mCD4 T cells incubated on SLBs either with ICAM-1-AF405 (200/μm^2) alone for 5 min or with ICAM-1-AF405 + I-E^k-MCC (2, 20 or 200/μm^2) for 20 min, labelled with anti-mouse TCRβ (cyan) and anti-CHC (magenta). N_{cells} > 34 per density. Scale bar, 5 μm. Right panels are quantifications of the MFI of TCRβ and CHC across the synaptic interface. Lines are median value +/- IQR and cyan dots are average values from individual experiments. **B** Representative TIRF-SIM micrographs of hCD4 T cells incubated on SLBs with ICAM-1-AF405 (200/μm^2) + anti-CD3ε UCHT1-AF488 (cyan, 30/μm^2) for the indicated times, labelled with anti-CHC (magenta). The right panel is quantification of the temporal PCC between CHC and CD3ε across the synaptic interface. N_{cells} ≥ 56. Scale bar, 5 μm.

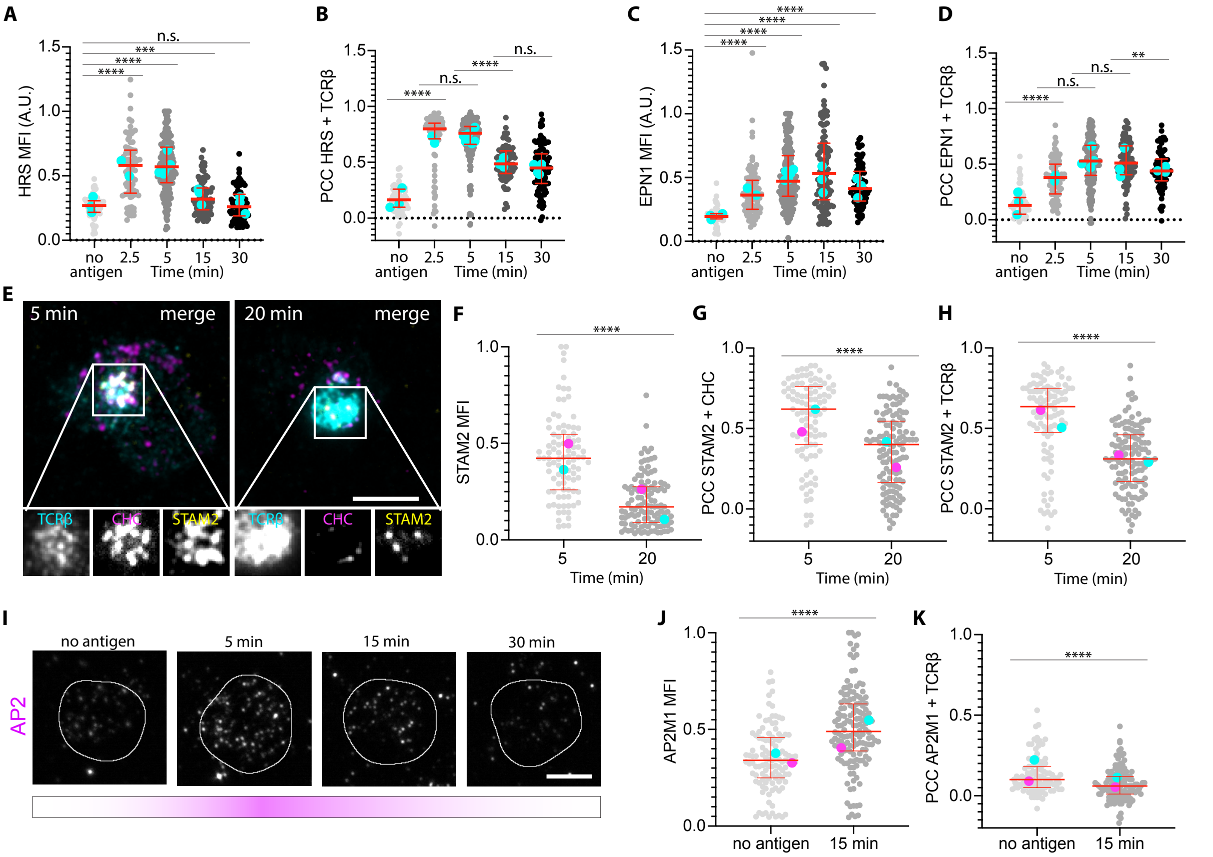

Fig. S2. HRS, EPN1 and STAM2 are recruited to TCR microclusters, but not AP2. A-B Quantification of the temporal MFI of HRS (**A**) and the temporal PCC between HRS and TCRβ (**B**) across the synaptic interface from the micrographs in Fig. 2A. Lines are median value +/- IQR and cyan dots are average values from individual experiments. **C-D** Quantification of the temporal MFI of EPN1 (**C**) and the temporal PCC between EPN1 and TCRβ (**D**) across the synaptic interface from the micrographs in Fig. 2B. **E** Representative TIRF micrographs of AND mCD4 T cells incubated on SLBs with ICAM-1-AF405 (200/μm^2) + I-E^k-MCC (20/μm^2) for 5 and 20 min, fixed, permeabilized and labelled with anti-TCRβ (cyan), anti-STAM2 (yellow) and anti-CHC (magenta). $N_{cells} \geq 94$ per timepoint. Scale bar, 5 μm. **F-H** Quantification of the temporal STAM2 MFI across the synaptic interface (**F**), the temporal PCC between STAM2 and CHC (**G**) and the temporal PCC between STAM2 and TCRβ (**H**). Lines are median value +/- IQR and cyan/magenta dots are average values from individual experiments. **I** Representative TIRF micrographs of AND mCD4 T cells incubated on SLBs either with ICAM-1-AF405 (200/μm^2) alone for 5 min or with ICAM-1-AF405 + I-E^k-MCC (20/μm^2) for 5, 15 and 30 min, labelled with anti-AP2M1. $N_{cells} \geq 87$ per timepoint. Scale bar, 5 μm. **J-K** Quantification of the temporal MFI of AP2M1 (**J**) and the temporal PCC between AP2M1 and TCRβ (**K**) across the synaptic interface. $N_{cells} \geq 105$ per timepoint.

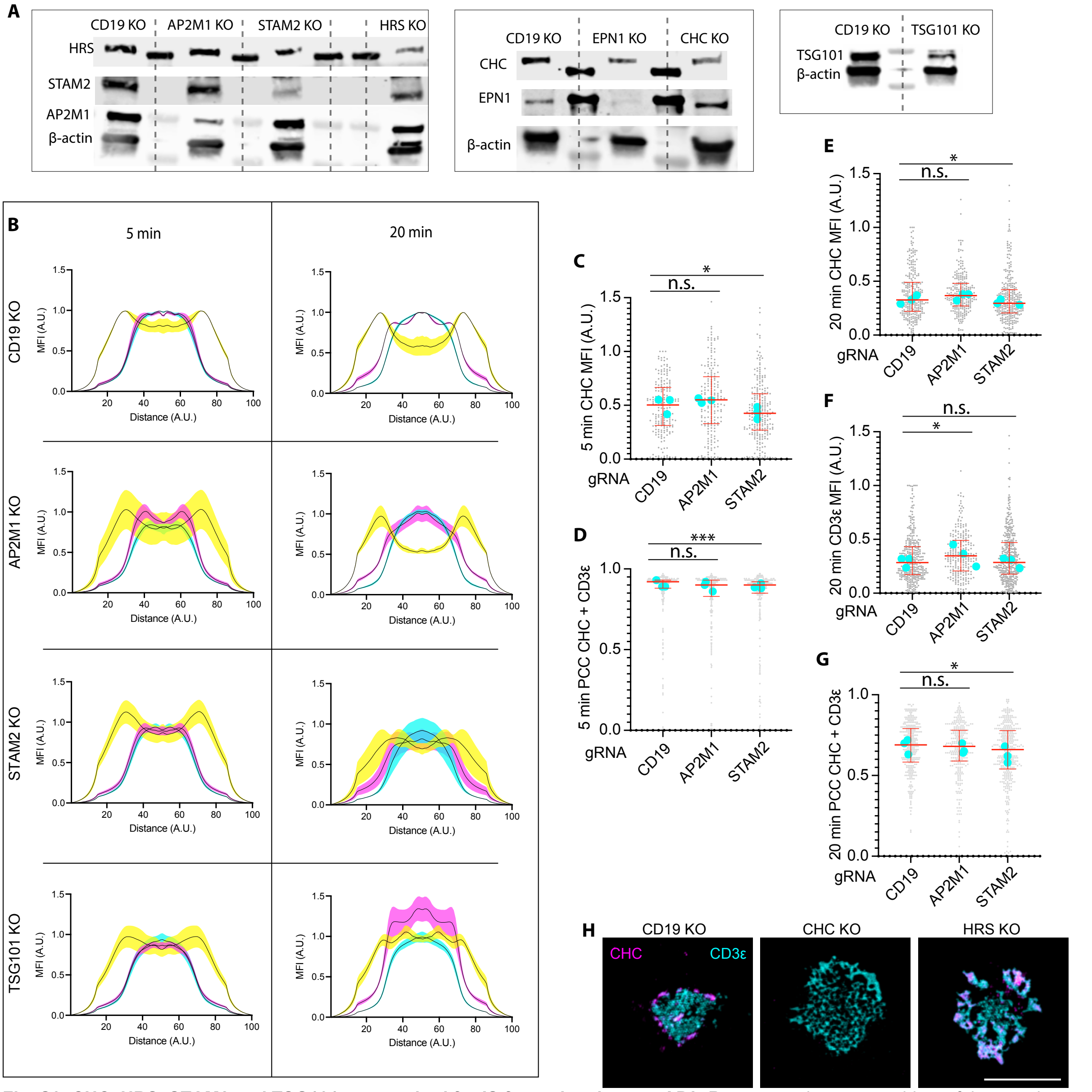

Fig. S3. CHC, HRS, STAM2 and TSG101 are required for IS formation, but not AP2. Representative western blots of the protein levels of CHC, HRS, EPN1, STAM2, AP2 and TSG101 following CRISPR/Cas9-mediated KO of the respective proteins. Anti-β-actin was included as loading control. Dashed lines indicate protein standard. **B** Radial averages of hCD4 CD19, AP2, STAM2 and TSG101 KO T cells incubated on the SLBs from Figure 4B for 5 and 20 minutes. MFI represents mean fluorescence intensity from 3 experiments +/- SEM. **C-G** Quantification of the MFI of CHC (**C**, **E**), CD3ε (**F**) and the PCC between CHC and CD3ε (**D, G**) across the synaptic interface from hCD4 CD19, AP2 and STAM2 KO hCD4 T cells incubated on the SLBs from Fig. 4B for 5 and 20 minutes, respectively. $N_{cells} \geq 165$ per condition. Lines are median value +/- IQR and cyan dots are median values from individual experiments. **H** TIRF-SIM micrographs of hCD4 CD19, CHC and HRS KO T cells incubated on the SLBs from Fig. 4B. Scale bar, 5 µm.

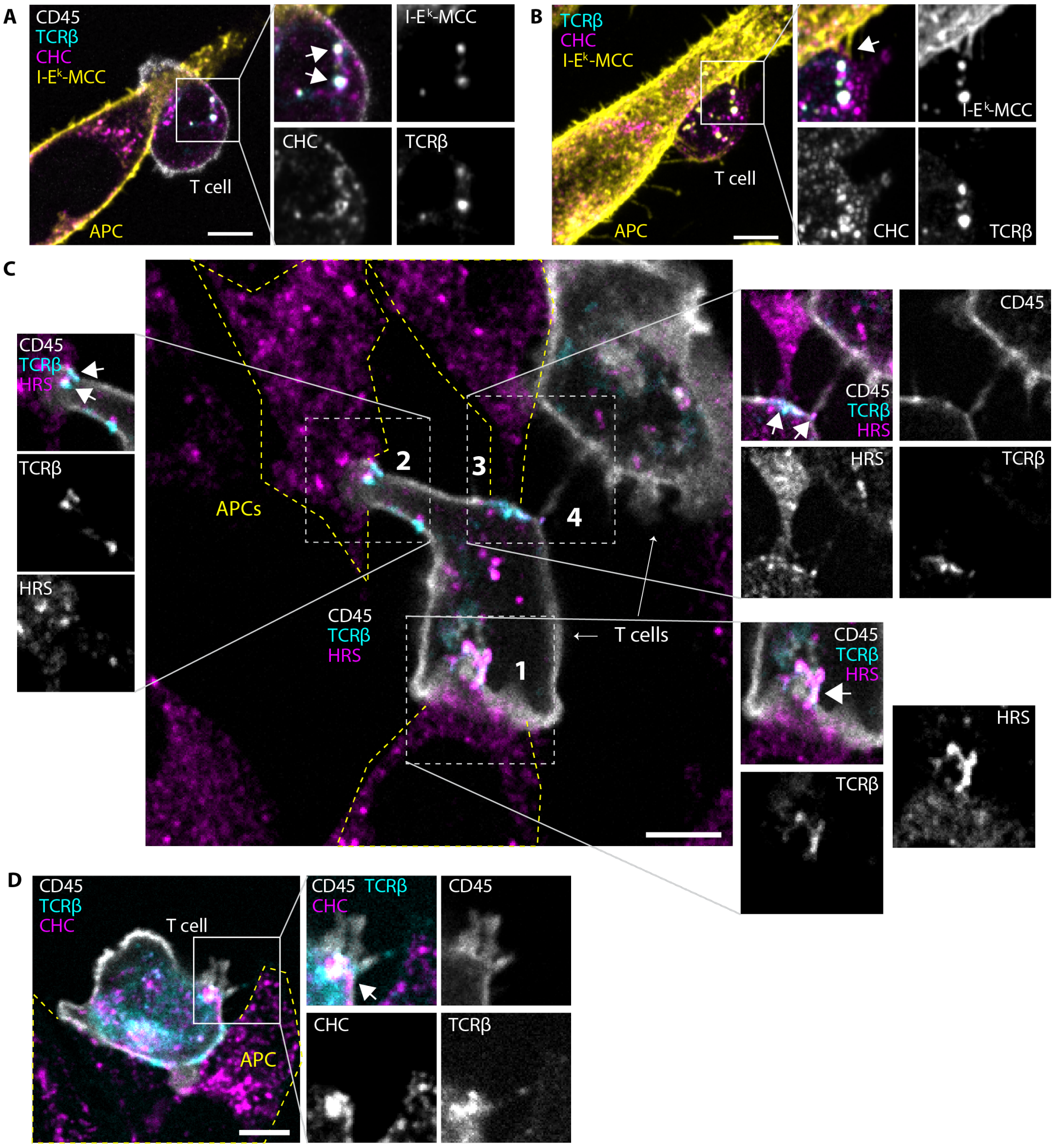

Fig. S4. Clathrin and HRS regulate microvillar membrane transfer. A Slice from an Airyscan® Z-stack with a step size of 250 nm of an mCD4 AND T cell incubated with CHO-I-E^k antigen presenting cells for 30 min and immunolabelled with anti-CD45 (white), anti-TCRβ (cyan), anti-CHC (magenta) and anti-I-E^k-MCC (yellow). White arrows indicate endosomal structures with overlapping I-E^k-MCC, TCRβ and CHC. **B** is a maximum intensity projection of the Z-stack from A emphasizing the microvillar protrusions from the APC. White arrow indicates CHC overlapping with I-E^k-MCC at the site of microvillar attachment. Scale bar, 5 µm. **C-D** A slice from a spinning disc confocal Z-stack with a step size of 250 nm of mCD4 AND T cells incubated with CHO-I-E^k APCs for 5 min and immunolabelled with anti-CD45 (white), anti-TCRβ (cyan), anti-I-E^k-MCC (yellow) and anti-HRS (**C**) or anti-CHC (**D**) (magenta). The intensities in some of the cropped images has been adjusted for emphasis. Yellow dashed lines indicate outlines of APCs. White arrows indicate overlap between TCRβ and HRS (**C**) or CHC (**D**). Scale bar, 5 µm.

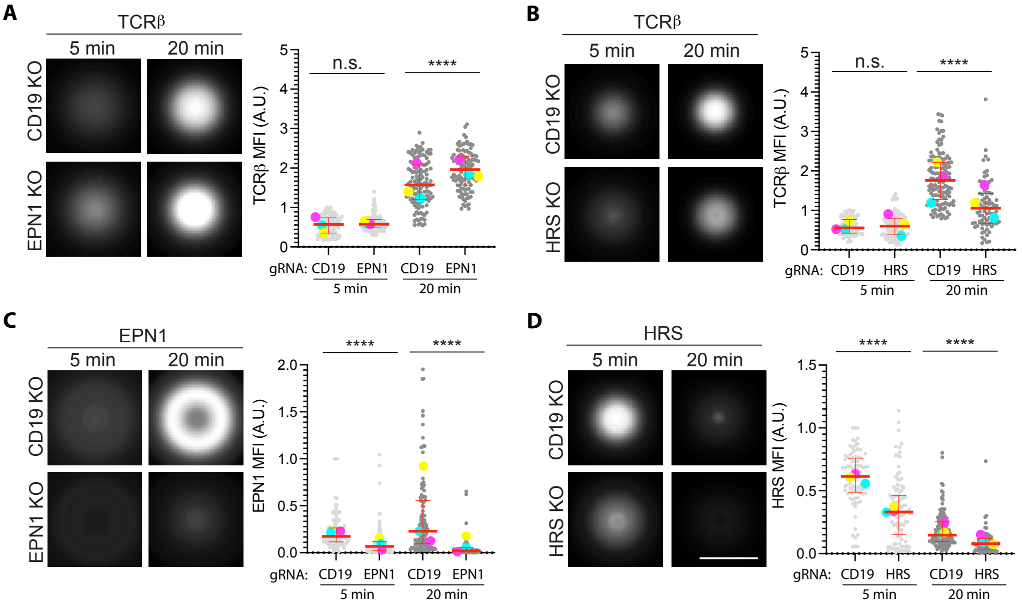

Fig. S5. EPN1 is required for TCR removal from the cSMAC while HRS is required to accumulate it there. A-D Radial averaged micrographs of segmented cSMACs from one representative experiment of CD19, EPN1 and HRS KO AND T cells incubated on SLBs with ICAM-1-AF405 (200/μm^2) and I-E^k-MCC (20/μm^2) for 5 and 20 minutes. The right panels are quantifications of the MFI of TCRβ, EPN1 or HRS within the cSMAC. $N_{cells} \geq 107$ per timepoint. Scale bar, 2.5 μm. Lines are median value +/- IQR and cyan/magenta/yellow dots are average values from individual experiments.

Movie S1. Clathrin is recruited to TCR microclusters. Representative movie of an AND T cell expressing CLCa-mCherry (magenta) incubated on an SLB for 5 min with ICAM-1-AF405 (200/μm^2) and I-E^k-MCC (50/μm^2). The TCR is labelled with anti-TCRβ (cyan). The framerate is 0.3 fps.
